# Dehydrofluorination Process of Poly(vinylidene difluoride) PVdF-Based Gel Polymer Electrolytes and Its Effect on Lithium-Sulfur Batteries

**DOI:** 10.3390/gels9040336

**Published:** 2023-04-14

**Authors:** Julen Castillo, Adrián Robles-Fernandez, Rosalía Cid, José Antonio González-Marcos, Michel Armand, Daniel Carriazo, Heng Zhang, Alexander Santiago

**Affiliations:** 1 Centre for Cooperative Research on Alternative Energies (CIC EnergiGUNE), Basque Research and Technology Alliance (BRTA), 01510 Vitoria-Gasteiz, Spain; 2 Department of Chemical Engineering, Faculty of Science and Technology, University of the Basque Country UPV/EHU, Campus de Leioa, Barrio Sarriena, 48940 Leioa, Spain; 3 IKERBASQUE, Basque Foundation for Science, 48013 Bilbao, Spain; 4 Key Laboratory of Material Chemistry for Energy Conversion and Storage (Ministry of Education), School of Chemistry and Chemical Engineering, Huazhong University of Science and Technology, Wuhan 430074, China

**Keywords:** lithium-sulfur battery, gel polymer electrolyte, dehydrofluorination, poly(vinylidene difluoride)

## Abstract

Gel polymer electrolytes (GPEs) are emerging as suitable candidates for high-performing lithium-sulfur batteries (LSBs) due to their excellent performance and improved safety. Within them, poly(vinylidene difluoride) (PVdF) and its derivatives have been widely used as polymer hosts due to their ideal mechanical and electrochemical properties. However, their poor stability with lithium metal (Li^0^) anode has been identified as their main drawback. Here, the stability of two PVdF-based GPEs with Li^0^ and their application in LSBs is studied. PVdF-based GPEs undergo a dehydrofluorination process upon contact with the Li^0^. This process results in the formation of a LiF-rich solid electrolyte interphase that provides high stability during galvanostatic cycling. Nevertheless, despite their outstanding initial discharge, both GPEs show an unsuitable battery performance characterized by a capacity drop, ascribed to the loss of the lithium polysulfides and their interaction with the dehydrofluorinated polymer host. Through the introduction of an intriguing lithium salt (lithium nitrate) in the electrolyte, a significant improvement is achieved delivering higher capacity retention. Apart from providing a detailed study of the hitherto poorly characterized interaction process between PVdF-based GPEs and the Li^0^, this study demonstrates the need for an anode protection process to use this type of electrolytes in LSBs.

## 1. Introduction

In the current global energy context, in which energy demand is continuously growing, replacing widely used fossil fuels with renewable and sustainable energy sources is a must. The development of efficient energy storage systems plays a pivotal role in driving this necessary transition. Lithium-ion batteries (LIBs) have become the most developed electrochemical energy storage technology and have largely dominated the market since their commercialization in the early 1990s by SONY [1,2,3]. However, the state-of-art (SoA) LIBs are reaching their practical limit, being unable to meet the requirements of high energy density applications, e.g., long-range electric vehicles or aviation [4,5,6]. In this scenario, where a novel battery electrochemistry is needed, lithium-sulfur batteries (LSBs) have emerged as appealing alternatives for replacing LIBs in some specific applications driven by the low cost, environmental friendliness, and the high theoretical capacity of the sulfur active material [7,8,9]. Despite these promising features, the practical performance and, hence, the wide commercialization of LSBs have been compromised by several key challenges, including the low sulfur utilization, high cathode volume change during cycling, lithium polysulfide (LiPS) shuttle effect, and severe anode degradation [10,11,12].

Traditionally, liquid electrolytes based on organic solvents have been extensively employed for LSBs, mainly based on ether-type compounds, through the classical mixture of 1,3-dioxolane (DOL)/1,2-dimethoxyethane (DME) [13]. Nevertheless, the use of this type of electrolyte entails several safety issues such as leakage, short-circuit, and combustion issues due to its highly flammable nature [14,15]. Given the excellent characteristics that the lithium metal (Li^0^) anode brings to the development of future high-energy density batteries, replacing the flammable liquid electrolytes with safer alternatives is of key importance to ensure the safety of the battery system [16,17]. At this point, the replacement of liquid electrolytes by all solid-state electrolytes (ASSE) emerges as one of the most promising solutions, since it could simultaneously solve all the safety issues regarding flammability, preventing dendrite formation, and diminishing the LiPS shuttle effect [18,19].

Solid polymer electrolytes (SPEs) have been extensively studied in this field due to their low cost, mechanical flexibility, and excellent large-scale manufacturing processability [20,21,22]. However, their low ionic conductivity at room temperature is the main obstacle to their final application, especially for the most typically studied SPE, polyethylene oxide (PEO), as it is required to work above its melting temperature of 65 °C [23,24]. One interesting approach to overcome the poor ionic conductivity of PEO-based SPEs at low temperatures is the incorporation of a plasticizer within the solid polymer matrix, forming a gel polymer electrolyte (GPE) [25,26,27]. This incorporation allows for developing electrolytes that combine both the advantages of liquid electrolytes, i.e., high conductivities and low interfacial resistances, and also the benefits of SPEs in terms of mechanical strength and thermal stability [28,29], whilst several strategies have been carried out to improve GPEs performance by taking advantage of these properties [30]. In GPEs, the high ionic conductivity is not mainly due to the ion movement through the long-chain polymer, but rather to the transport of solvated molecules, and it is therefore associated with the plasticizer component [31].

Different polymers have been used for developing GPEs [32], but poly(vinylidene difluoride) (PVdF) and its derivatives stand out among the rest due to their suitable individual properties, including a high dielectric constant, easy processing, and excellent thermal and electrochemical stability (wide voltage window), although interfacial stability issues due to the interaction with Li^0^ anodes have also been reported [33,34,35]. Among PVdF-based GPEs, the copolymer formed with hexafluoropropylene (PVdF-HFP) exhibits excellent properties such as improved thermal stability and a higher dielectric constant, in part because the disorder added to the system leads to higher amorphocity resulting in higher plasticizer uptake, hence leading to an improvement in the ionic conductivity [34]. Nevertheless, there is a considerable controversy surrounding the cathodic stability of PVdF-based polymers in lithium metal batteries (LMBs) [36]. There is a consensus in the literature regarding the reactivity of these polymers with the Li^0^ anode, but the potential benefits or harms that this process may cause to cell stability have not completely been addressed [34,37,38,39,40]. The reaction between Li^0^ and fluorine atoms leads to the dehydrofluorination of PVdF on the—CH_2_CF_2_—segments and the reduction of the vicinal fluorides on the HFP moieties to the formation of a LiF-rich solid-electrolyte interphase (SEI) on the surface of the Li^0^ anode. Assuming this reactivity between the polymer and the Li^0^ anode, some works state the unsuitability of fluorine-based polymers for the fabrication of LMBs [40,41,42,43], and other reports highlight that the formation of this LiF layer prevents the further corrosion of the Li^0^ anode, thus providing higher stability [44,45]. The dehydrofluorination process of PVdF has been specially analyzed when combined with a garnet-type ceramic for the preparation of composite polymer electrolytes (CPEs), a phenomenon that becomes exacerbated when coupled with a Li^0^ anode [46,47]. In these cases, the dehydrofluorination of PVdF is driven by alkaline-like conditions induced by certain garnets, e.g., LLZTO. However, Bag et al. [41] reported the degradation of a PVdF-based CPE after cycling with a Li^0^ anode leading to the dehydrofluorination of the polymer, which they considerably solved by incorporating LiF as an additive, thus successfully stabilizing the system.

Hence, the reactivity of PVdF-derived polymers with the Li^0^ anode has always been considered when describing the electrochemical features and properties of these polymers. However, few works have studied in depth the origin of this degradation, the effects that these dehydrofluorination processes may have on the final battery performance, and the existing differences depending on the type of battery chemistry selected. Encouraged by this fact and following the previous study carried out by our group [48], in this paper a comprehensive study of PVdF-HFP-based gel polymer electrolytes plasticized with poly(ethylene glycol dimethyl ether) (PEGDME_GPE), and tetraethylene glycol dimethyl ether (TEGDME_GPE), for their application in LSBs is presented. In this case, the electrochemical behavior of these GPEs is addressed by pairing with both Li^0^ symmetric and lithium-sulfur cells, putting effort into detailing the polymer host dehydrofluorination process induced by the interaction with Li^0^ and the possible effect that the defluorinated membrane could experience when exposed to the presence of LiPS regarding cell stability and electrochemical performance.

## 2. Results and Discussion

**Figure 1:**
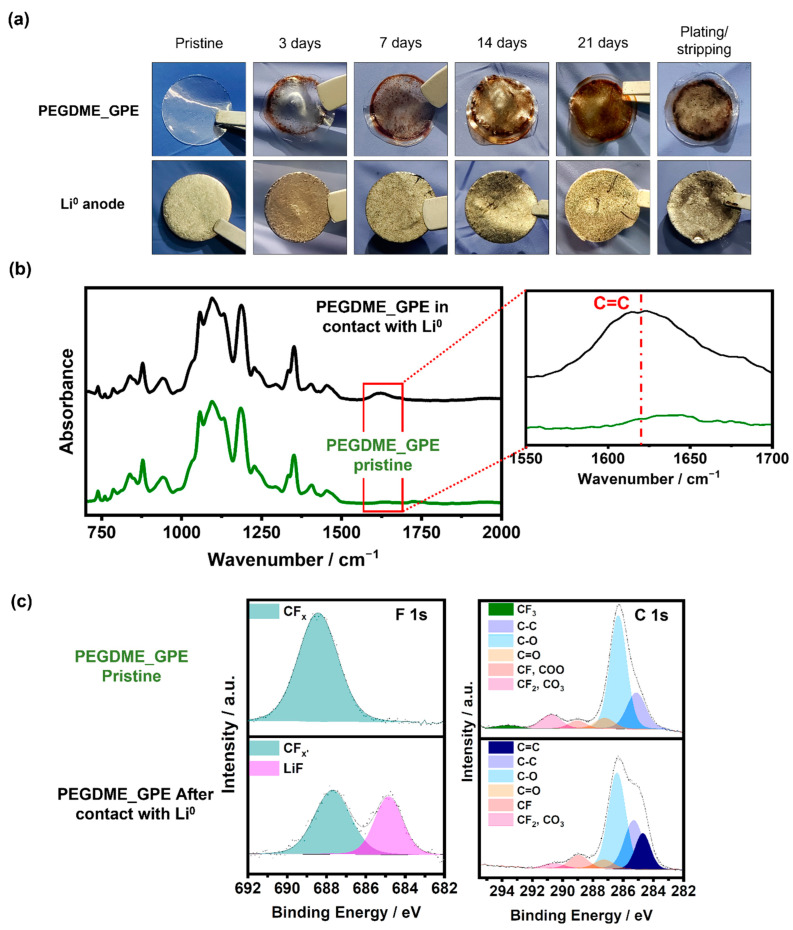
(**a**) Aging test of PEGDME_GPE in contact with Li^0^ anode. (**b**) ATR-FTIR spectra of the PEGDME_GPE-based electrolyte before and after being in contact with Li^0^ anode. (**c**) F 1s and C 1s regions corresponding XPS of the pristine PEGDME_GPE membrane and PEGDME_GPE after contact with Li^0^ anode.

PVdF and some of its derivatives, including PVdF-HFP, have been widely studied as polymeric matrices in gel polymer electrolytes. This material was selected due to its good mechanical properties, thermal stability, and good retention of liquid electrolytes within. This was the case of the gel electrolyte developed in this work, which incorporated 80 wt.% of liquid electrolyte while maintaining its structural integrity. In general, fluorinated polymers are highly resistant to electrochemical degradation, but although several studies claimed that their stability is penalized under basic conditions, which could be associated with their reactivity with the Li^0^, very few studies have investigated it. Therefore, to confirm the chemical stability of the membranes developed in this work, different lithium-symmetric cells were assembled and stored for several days for their post-mortem analysis. The results of this study are shown in Figure 1a. This figure reveals the instability of the electrolyte against lithium, as it can be claimed from the color changes observed over time and the evolution of brownish areas. The colored zones increase with exposure time, indicating that a chemical reaction between the Li^0^ and the GPEs is taking place. Furthermore, this reaction occurs regardless of the plasticizer used to develop the gel electrolyte, as pointed out by the brownish areas in both electrolytes, but the degree of reaction between the Li^0^ anode and the TEGDME_GPE is much lower, as depicted in Figure S1. The stability of the plasticizer against lithium has also been demonstrated by the substitution of the polymer matrix. Figure S2 shows a similar stability study carried out using electrolytes where PVdF-HFP is replaced by poly(methyl methacrylate) (PMMA). As can be observed, no reaction occurs in this case and the membrane remains transparent, regardless of the time. With the aim of confirming the stability of the liquid electrolyte component with the Li^0^ anode, the same stability test was carried out with a Celgard^®^ separator. As can be appreciated in Figure S3, both the Li^0^ anode and the separator remain unaltered.

To find out the reason behind this color change, the FTIR and XPS measurements were carried out. The results are presented in Figure 1b, and Figure 1c, respectively, for PEGDME_GPE and Figure S4 for the case of TEGDME_GPE. For the infrared analysis, the pristine polymer gel was taken as a reference and compared with its counterpart after seven days in contact with Li^0^. As can be seen in Figure 1b, although there is no significant variation in the spectra, in addition to the characteristic bands associated with the plasticizer observed at 1300 cm^−1^ (referred to CH_2_) and at 1100 cm^−1^ (ascribed to C–O–C groups) and those related to the polymer host (1400 and 1200 cm^−1^ for CH_2_ cm^−1^ and CF_2_, respectively), a band registered at 1600 cm^−1^ evolves. This signal can be ascribed to the C=C double bond formation due to the reactivity of the polymer host [49,50,51], since it is formed independently of the plasticizer used in the electrolyte (Figure S4). To confirm this finding, an XPS analysis was also carried out. Figure 1c includes the XPS of both pristine gels and after storage in contact with lithium. The F 1s spectrum of the pristine one shows a broad peak centered at 688.4 eV, revealing the presence of CF_x_ units. Its broadness comprises the three CF_3_, CF_2,_ and CF chemical environments of the PVdF-HFP, since little chemical shift exists among them in the F 1s core level. However, the chemical shift among the different CF_x_ environments is very pronounced in the C1s spectrum and well separated peaks are found at 293.7 eV, 290.8 eV, and 289.0 eV for CF_3_, CF_2,_ and CF units, respectively, due to the noticeable reduction in the electron density of C atoms with the increasing number of highly electronegative F neighbors. Nevertheless, after contact with Li^0^, the peak at 293.7 eV (CF_3_) disappears and the one at 290.8 eV (CF_2_) is also reduced, while the signal at 289.0 eV (CF) increases, clearly indicating the defluorination of the gel electrolyte in contact with Li^0^. This agrees with the shift and narrowing of the CF_x_ component observed in the F 1s spectrum (now peaked at 687.7 eV). Moreover, part of the F in the gel has reacted with the Li^0^ leading to the formation of LiF in the interface (peak at 684.8 eV). In addition, the signal in the low binding energy (BE) part of the C 1s spectrum increases and an extra component at 284.5 eV is needed to correctly fit the signal, indicating the formation of C=C bonds as already suggested by FTIR.

As a summary, the preliminary results shown in Figure 1 point out that the chemical instability of the PVdF can be explained by the dehydrofluorination reaction. This reaction gives rise to the release of HF and the formation of C=C double bonds (crosslinking between chains), which is promoted by the alkaline environment generated by the native solid electrolyte interface layer of the Li^0^ rich in carbonates.

**Figure 2:**
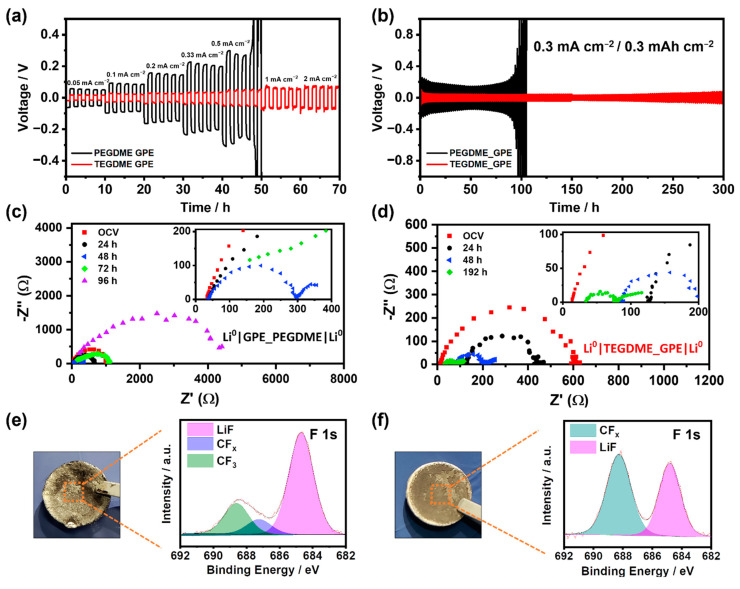
(**a**) Galvanostatic cycling of Li^0^ symmetric cells using PEGDME-based (black) TEGDME-based (red) electrolytes under different current densities (from 0.05 mA cm^−2^ to 2 mA cm^−2^) and with 1h for the half cycle. (**b**) Galvanostatic cycling of Li^0^/PEGDME_GPE/Li^0^ (black) and Li^0^/TEGDME_GPE/Li^0^ (red) cells at 0.3 mA cm^−2^ and 0.3 mAh cm^−2^. (**c**) Electrochemical impedance spectroscopy (EIS) over time of Li^0^/PEGDME_GPE/Li^0^ (**d**) EIS over time of Li^0^/TEGDME_GPE/Li^0^. (**e**,**f**) Optical images and F 1s region corresponding XPS of the Li^0^ anode after the galvanostatic cycling with PEGDME_GPE and TEGDME_GPE, respectively.

Good electrochemical compatibility between the Li^0^ anode and GPE is of paramount importance for the optimum performance of the LMBs since it guarantees the long-term cyclability of the system. To evaluate this compatibility, different galvanostatic cycling tests of Li^0^||Li^0^ symmetric cells have been carried out at room temperature. First, a current test was performed using both GPEs at different current densities from 0.05 mA cm^−2^ to 2 mA cm^−2^ (Figure 2a). As can be observed in Figure 2a, although the PEGDME_GPE shows a low overpotential and smooth performance, the maximum critical current density supported is 0.33 mA cm^−2^. At higher values, the cycling becomes unstable, hindering cell cyclability. On the other hand, the Li^0^ symmetric cell with TEGDME_GPE displays a lower overpotential and more stable cycling in the whole current steps in comparison with the performance offered by the PEGDME_GPE cell, especially under high current densities showing an incredibly low overpotential of 70 mV at 2 mA cm^−2^.

Afterward, considering the maximum current density supported by both electrolytes, constant galvanostatic cycling was performed at 0.3 mA cm^−2^ current density and 0.3 mAh cm^−2^ areal capacity. As displayed in Figure 2b, in the case of PEGDME_GPE, stable cycling with a 200 mV overvoltage for more than 90 h has been achieved. After that cycling time, its overpotential fluctuated and progressively increased until the eventual cell failure. This behavior can be attributed to the formation of an excessively thick layer as a consequence of its interaction with the electrolyte, thus increasing the resistivity and eventually hindering Li^+^ diffusion. On the other hand, TEGDME_GPE presents smoother profiles and improved stability with a lower overpotential of 40–50 mV for more than 300 h. Likewise, there is no presence of erratic cycles or evidence of dendrite-caused short circuits during cycling, as evidenced in Figure S6. The lower overpotential in both galvanostatic tests can be ascribed to the remarkably higher conductivity of TEGDME_GPE, as proven in Figure S5. It should be noted that none of the cells went into short-circuit, with the PEGDME cell failing after ~100 h due to an exponential increase in overvoltage. This circumstance underlines the importance of the LiF-rich SEI layer to inhibit the growth of lithium dendrites [33].

To get insight into the behavior of both electrolytes during the galvanostatic cycling at 0.3 mA cm^−2^, electrochemical impedance tests of the symmetric cells were carried out after each set of five plating and stripping cycles. As shown in Figure 2c,d, Nyquist plots of PVdF-HFP-based cells exhibit different behavior depending on the plasticizer used to generate the GPE. In the case of the PEGDME_GPE cell (Figure 2c), the bulk resistance R_b_ associated with lithium-ion transport through the membrane barely changes during the experiment. On the other hand, the TEGDME_GPE cell (Figure 2d) shows different electrochemical behavior. Lower initial bulk resistances R_b_ are found in comparison to that of the PEGDME_GPE cell, indicating a higher conductivity of the TEGDME_GPE as previously shown in Figure S5. Nevertheless, the R_b_ increases in the first galvanostatic cycles, indicating a lowered Li^+^ mobility through the GPE when passing a moderate current of 0.3 mA cm^−2^ across the cell. Afterward, bulk resistance of the TEGDME_GPE cell slowly diminishes at the point of even reaching the initial values measured before cycling, as shown on the inset of Figure 2d.

Focusing now on the interfacial resistance, the semicircle registered for the PEGDME_GPE ostensibly changes. First, it progressively decreases up to 50 h of cycling at 0.3 mA cm^−2^, which is consistent with the lowered overvoltage of the cell shown in Figure 2b. This could be explained through the benefits derived from the stable LiF passivating layer formation over the Li^0^ electrode and the initially improved kinetics of the interfacial Li^+^ transport. Beyond 50 h of cycling, the interfacial resistance progressively increases, mainly due to the GPE membrane degradation through the dehydrofluorination process and the creation of a thicker and rough surface in the Li^0^ anode [52]. These impedance data are also consistent with the abrupt overvoltage increase observed at ~100 h in Figure 2b. On the other hand, in the TEGDME_GPE cell interfacial resistances decrease continuously with cycling time and stabilize, but they do not show signs of increasing, at least for the first 300 h of the galvanostatic cycling at 0.3 mA cm^−2^. Taking a look at the overvoltage profile of the TEGDME_GPE cell in Figure 2b, this is the expected behavior due to the lack of clear signs of membrane degradation throughout the experiment.

To further analyze the galvanostatic cycling performance behavior in the symmetric cells of both prepared GPEs, XPS measurements of the Li^0^ anodes were carried out (Figure 2e,f). In both cases, it is shown that dehydrofluorination of the polymer matrix results in the generation of a LiF-rich SEI layer, which, as mentioned above, is of paramount importance to suppress the lithium dendrites growth due to its good mechanical properties. However, in the case of the PEGDME-based GPE (Figure 2e) the presence of LiF is much more pronounced, leading us to assume that the generated layer is much thicker than in the case of the TEGDME-based GPE (Figure 2f), which would explain the increase of the overvoltage in the system due to the lower ionic conductivity of that compound.

**Figure 3:**
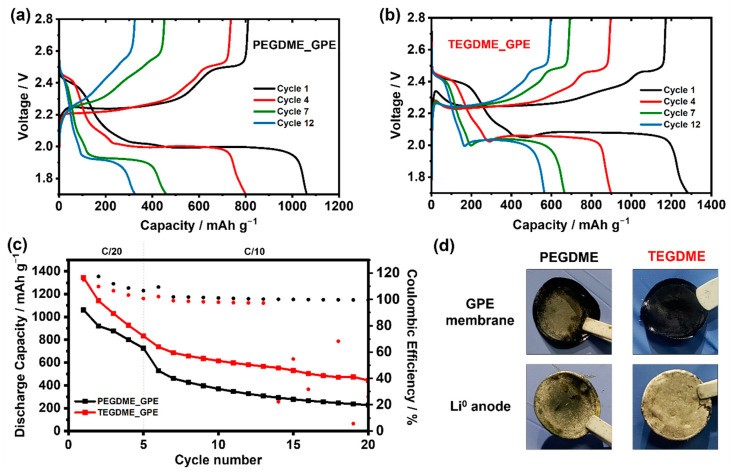
(**a**,**b**) Charge/Discharge profiles for PEGDME_GPE and TEGDME_GPE Li-S cells, respectively. (**c**) battery performance of developed GPEs. (**d**) Optical post-mortem images of the analyzed GPEs and their corresponding Li^0^ anode.

The high reactivity of PVdF-based GPEs with the Li^0^ anode has been proved just by their contact without cycling and more clearly evidenced upon galvanostatic cycling. However, the stability observed in the galvanostatic cycling and the results obtained in our previous work [48] using this type of GPEs reveal that this reactivity leads to improved stability of Li^0^-based systems, owing to the formation of a LiF-rich SEI layer. To the best of our knowledge, this kind of GPE has not been tested in LSBs and, consequently, neither stability nor performance have been evaluated in this battery technology. Thus, the applicability of the two GPEs prepared in this work has been evaluated in LSBs cycling at room temperature, as shown in Figure 3. The discharge/charge profiles at C/20 (cycles 1 and 4) and at C/10 (cycles 7 and 12) of both GPEs are presented in Figure 3a,b, displaying the two characteristic plateaus of the sulfur redox reactions taking place in a Li-S battery. Notably, the PEGDME_GPE cell shows a higher overvoltage between the charging and discharging processes, which becomes significantly higher as the cycling rate increases (cycles 7 and 12) compared to the overvoltage shown by the TEGDME_GPE cell. This clear difference of both electrolytes is directly related to the lower ionic conductivity of PEGDME_GPE concerning TEGDME_GPE due to the higher viscosity of the plasticizer.

In terms of cycling performance (Figure 3c), although the initial capacities of both GPEs are impressive and promising, delivering 1345 mAh g^−1^ for TEGDME_GPE and 1061 mAh g^−1^ for PEGDME_GPE, a dramatic capacity fade was observed in both studied electrolytes, resulting in a capacity of 400 mAh g^−1^ for TEGDME_GPE and 250 mAh g^−1^ for PEGDME_GPE after 20 cycles. These performance differences would be directly related to the higher ionic conductivity of TEGDME_GPE and its lower reactivity versus the Li^0^ anode.

**Figure 4:**
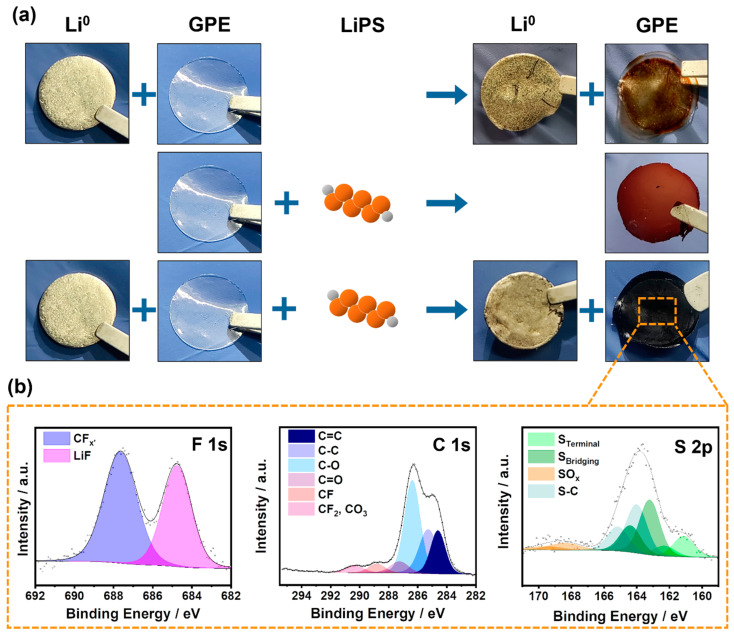
(**a**) Schematic illustration of the behavior of the membrane towards the different cell compounds. (**b**) XPS of F 1s, C 1s, and S 2p of the GPE after the combination of the dehydrofluorination process of the GPE and the presence of LiPS.

On the other hand, it is worth noting that during the first cycles, the obtained Coulombic efficiency is much higher than 100% in both cases. This behavior could have several plausible explanations such as the irreversibility of some of the polysulfide reactions or the loss of polysulfide intermediates into the electrolyte which would result in a progressive loss of active material in the cathode side. Therefore, a postmortem analysis was carried out to identify the cause among the different mentioned scenarios. As observed in Figure 3d, no additional degradation is observed in the Li^0^ anode in comparison with the one obtained after the galvanostatic cycling, implying that the drop in capacity is not related to any issue regarding the anode. In contrast, and regardless of the plasticizer used in the electrolyte composition, the membrane presents a dark color, which is more intense than in the case of lithium-symmetric cycled cells. The more intense degradation observed in the electrolytes of Li-S cells raises the question of the stability of the developed electrolytes to LiPS. For this purpose, the study was completed with a simple experiment as shown in Figure 4, which displays the compatibility of the gel polymer electrolyte when it is exposed to the different elements of the cell.

As described above, the electrolyte is not completely stable against the Li^0^ anode. By simple contact, a dehydrofluorination reaction takes place and part of the F belonging to the polymer matrix is removed while conjugated C=C bonds are formed in the polymer matrix, hence the observed brownish coloring on the surface of the polymer matrix. However, as it has been determined by the galvanostatic cycling, this dehydrofluorination does not harm the system. On the contrary, a positive effect due to the stabilization of the Li^0^ anode by the formation of a LiF-rich SEI layer is observed.

The stability of the system against LiPS was further studied. As can be seen in the attached image in Figure 4, although the membrane takes on a reddish hue due to the presence of these compounds, no degradation occurs in the electrolyte. Even so, it is worth noting from this experiment that the polymeric matrix will not act as a barrier to LiPS, as it is permeable. Finally, the interaction between the partially dehydrofluorinated matrix and the LiPS is evaluated. In this case, the matrix is completely degraded, showing a blackish color against the combined action of LiPS and dehydrofluorination. This interaction may be the reason for the observed dramatic loss of cycling capacity. As demonstrated above, the membrane is permeable to the LiPS generated during the discharge process and lost into the electrolyte. Moreover, due to the interactions between the carbon double bonds generated by the dehydrofluorination of the system and the LiPS species [49] these are retained within the electrolyte. This retention causes progressive leaching of the active material in the cathode, decreasing its capacity consequently. As can be seen in the XPS analysis of the sample (Figure 4b), the interaction between the LiPS and the polymeric matrix is of covalent character, generating C–S bonds (light blue S 2p doublet, which is needed to correctly fit the spectrum in addition to the two green doublets representing the bridging and terminal sulfurs of the LiPS chains). This reaction is similar to the one undergoing the vulcanization process, in which the sulfur groups react with the double bonds of the system.

**Figure 5:**
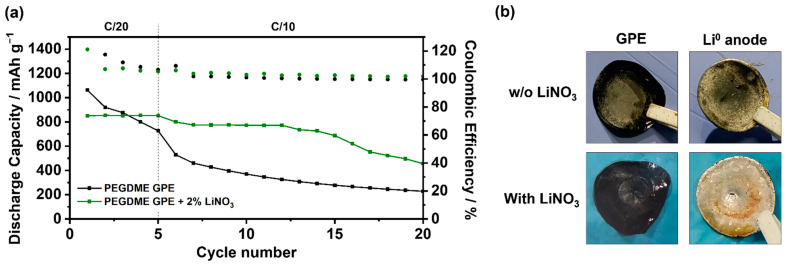
(**a**) Comparison of the battery performance of PEGDME_GPE with and without LiNO_3_ additive and (**b**) their corresponding optical post-mortem analysis of the membranes and Li^0^ anodes.

Considering the problems derived from the combination of the LiPS solution and the C=C bonds generated due to the reactivity of PVdF-based GPEs with the Li^0^ anode, the introduction of an additive that reduces both factors was proposed to improve the stability of the system. Hence, LiNO_3_ salt was selected given its ideal effect on the protection of the Li^0^ anode via spontaneous reaction and, although less well known, its role as an oxidative catalyst of LiPS by strongly binding them and facilitating their redox reactions [53,54]. Therefore, to improve the stability of the system, the PEGDME_GPE was modified by the addition of 2 wt.% of LiNO_3_. As depicted in Figure 5, the LSB performance of the two systems (with and without the LiNO_3_ additive) was compared, revealing a clear improvement in cell performance after the LiNO_3_ addition. The initial capacity is slightly lower in the case of the LiNO_3_-containing cell, probably due to the additional resistance provided by the protective layer formed after the additive reduction on the Li^0^ anode surface. However, the clear improvement lies in the suppression of the constant capacity drop, characteristic in the two previously analyzed systems, displaying a clearly stable cycling with an acceptable discharge capacity value of 760 mAh g^−1^ after 15 cycles.

This clear improvement can be explained by two main reasons, both based on the reduction of reactivity between the C=C bonds and LiPS. On the one hand, the protective layer generated by LiNO_3_ on the surface of the Li^0^ anode prevents to some extent its reaction with the PVdF-based polymer host, thus reducing its further reaction with the LiPS. On the other hand, owing to its ability as a LiPS catalyst, LiNO_3_ allows retaining the generated LiPS at the cathode to a certain degree, favoring their redox conversion and reducing the migration of these LiPS, which would give rise to reactivity with the dehydrofluorinated GPE. It is noteworthy that the improvement obtained by LiNO_3_ is maintained solely during the first 15 cycles, from which the capacity fade was observed. This performance can be explained since the LiNO_3_ additive has been completely consumed. Indeed, this improvement highlights the importance and necessity of Li^0^ anode protection to overcome the problems previously studied in PVdF-based GPE in LSBs, either by introducing additives (as in this case) or by pretreatment of Li^0^ by ex-situ protection techniques. On the other hand, it also underlines the importance of LiPS retention at the cathode with the aim of removing its solubility into the electrolyte and thus avoid the undesirable reaction with the C=C bonds presented in the polymer host after the dehydrofluorination.

## 3. Conclusions

GPEs based on PVdF or its derivatives have been widely reported in the literature for their potential application in LMBs due to their outstanding features. In this work, an in-depth study of the commonly mentioned but understudied poor stability of PVdF-based GPEs with the Li^0^ anode has been carried out and their application in Li-S batteries has been further analyzed. The instability found is ascribed to a process known as dehydrofluorination, which occurs simply by the contact between Li^0^ and the membrane and is accelerated during cycling, as demonstrated. This process can be partially beneficial for LMBs since it results in the formation of a LiF-rich SEI layer, which provides high stability to the symmetrical lithium cells. However, in the case of LSBs, this process causes a negative effect due to the LiPS presence in the system which can interact with the formed C=C bonds of the reacted polymer matrix. These cells showed unsuitable cycling performances characterized by a constant capacity drop due to the consequent loss of active material. Nevertheless, it has also been proven that the incorporation of LiNO_3_ as an additive into the electrolyte formulation could reduce the interaction between the LiPS and the C=C bonds, resulting in a stable performance until the total additive consumption. These results demonstrate the importance of trapping the LiPS at the cathode to avoid possible reactions either with the Li^0^ anode or with other cell components such as the electrolyte. In this way, our research sheds light on the poorly studied compatibility of PVdF-based GPEs with the Li anode, showing its negative effect on LSBs and proving the need for Li^0^ anode protection, either by in-situ or ex-situ protection techniques, as an indispensable step for the use of these electrolytes in Li-S technology.

## 4. Materials and Methods

### 4.1. Preparation of Gel Polymer Electrolytes

GPEs were prepared by a solvent casting method maintaining an average thickness of 80−100 μm for all the membranes. A 20:80 wt.% relation was kept constant between the PVdF-HFP polymer host and the plasticizer content, PEGDME or TEGDME. The following processes were involved for preparing the GPE membranes. First, the liquid electrolyte was prepared by dissolving stoichiometric amounts of battery grade lithium bis-(trifluoromethanesulfonyl)imide (LiTFSI 99.95%, Sigma-Aldrich, Madrid, Spain) into polyethylene glycol dimethyl ether (PEGDME 99% *M*_w_ = 500 g mol^−1^, Sigma-Aldrich, Madrid, Spain) or tetraethylene glycol dimethyl ether (TEGDME 99% *M*_w_ = 222 g mol^−1^, Sigma-Aldrich, Madrid, Spain), keeping an ethylene oxide/lithium molar ratio of 20:1. In parallel, the polymer host (PVdF-HFP *M*_w_~120,000 g mol^−1^, 2801 Kynar Flex, Arkema, Lacq/Mourenx, France) was dissolved in 4 mL of acetone. Once the liquid electrolyte and the polymer host solutions were prepared, they were mixed, stirring for 4 h at RT for homogenization. The obtained solution was cast thereafter in a polytetrafluoroethylene dish for solvent evaporation, which was carried out under vacuum to fully remove the remaining acetone from the obtained membrane.

For comparison, and following the same procedure, a GPE based on polymethyl methacrylate (PMMA *M*_w_~350,000 g mol^−1^, Sigma-Aldrich, Madrid, Spain) was prepared. In this case, due to the lower mechanical properties of the polymer host, the ratio between polymer and liquid electrolyte was modified to 40:60 wt.%.

### 4.2. Positive Electrode Preparation

The composition of the S@KJ600-ResFArGO sulfur-positive electrode used on lithium-sulfur gel polymer electrolyte cells comprises 64 wt.% sulfur (S_8_, ≥99%, Sigma-Aldrich, Madrid, Spain), 26 wt.% of carbonaceous materials, and 10 wt.% of binder compounds. The carbons used in this electrode are 16 wt.% of commercial Ketjenblack^®^ EC-600JD (KJ600, Nouryon, Barcelona, Spain) and 10 wt.% of an in-house made graphene-based activated carbon, ResFArGO, whose synthesis and properties are detailed elsewhere [55]. In order to ensure good intimate contact between the active material and the conductive carbons, a melt–diffusion process at 155 °C for 12 h was carried out on the mixture to infiltrate sulfur into the different carbons. As for the binders, 5 wt.% of carboxymethylcellulose sodium salt (CMC, Sigma-Aldrich, Madrid, Spain) and 5 wt.% of styrenebutadiene rubber (SBR, Jingrui, Shanghai, China) were used. Distilled water was used as solvent for the manufacture of this electrode. The cathode slurry was cast on a carbon-coated aluminum current collector by the Doctor Blade technique. 13 mm diameter cathodes were then punched, achieving low loading cathodes of around ~1.5 mg_s_ cm^−2^, used for the long-cycling tests.

### 4.3. Electrochemical Tests

Coin cells were assembled inside an argon-filled glove box. Li^0^|GPE|Li^0^ symmetrical CR2032 cells were prepared with the GPE sandwiched between two 500 μm thick lithium metal disks (China Energy Lithium). For the aging tests, these cells were stored in the glove box and opened 3, 7, 14, and 21 days after their preparation.

Galvanostatic cycling tests were performed over symmetrical cells, testing from 0.05 mA cm^−2^ up to 2 mA cm^−2^ and increasing the current density every 5 complete cycles with a half-cycle duration of 1 h. Long-cycling plating and stripping tests were performed by galvanostatic cycling of the symmetrical cells at current densities of 0.1 mA cm^−2^ and 0.3 mA cm^−2^ with a half-cycle duration of 1 h using a Maccor Battery Tester (Series 4000). For the electrochemical impedance spectroscopy (EIS) tests, the same cycling conditions were applied to the symmetrical cells using a VMP3 potentiostat (BioLogic, Seyssinet-Pariset, France). AC impedance tests were performed before galvanostatic cycling and then every 5 plating and stripping cycles (10 h), sweeping between 10^5^ Hz and 10^−2^ Hz with a V_AC_ amplitude of 10 mV. Impedance data were fitted to *R_b_* + *R_1_/Q_1_* equivalent circuits, where *R_b_* is the resistance associated with Li^+^ transport through the membrane and *R_1_/Q_1_* is a resistance placed in parallel with a constant-phase element, which represents the electrochemical phenomena taking place at the Li^0^/GPE interfaces.

CR2032 conductivity cells were assembled with 8 mm diameter GPE membranes and a 16 mm diameter Kapton ring-shaped film in which the GPE was embedded. Two stainless steel disks acted as blocking electrodes for the EIS measurements. AC impedance tests were performed using a VMP3 potentiostat (BioLogic, Seyssinet-Pariset, France) in the frequency range from 10^6^ Hz to 10^−1^ Hz with a voltage amplitude of 10 mV. From these measurements, the *R_b_* value can be withdrawn, and the ionic conductivity *σ* of the membrane is calculated using the following expression, where *l* is the membrane thickness and *S* is the GPE surface (0.503 cm^2^):

(1) $$\sigma =\frac{l}{R_{b}\cdot S}\;$$

Lithium-sulfur cells were assembled with the aforementioned S@KJ600-ResFArGO cathode and 500 μm thickness Li^0^ anode in CR2032-type cells. Li-S cells were galvanostatically cycled in a Maccor Battery Tester (Series 4000) at C/10 with 5 preconditioning cycles at C/20, between 1.7 and 2.8 V cut-off voltage range.

### 4.4. Physicochemical Characterization

X-Ray photoelectron spectroscopy (XPS) measurements were carried out on the surface of the GPE membrane and Li^0^ anode. The former was measured as-prepared, then after the aging tests in contact with Li^0^, after galvanostatic cycling, and after cycling in a Li-S cell. The latter was measured in order to prove the existence of a LiF-rich SEI layer generated by the PVdF-HFP contact with the Li^0^ electrode. All measurements were carried out in a Phoibos 150 XPS spectrometer (SPECS Surface Nano Analysis) operated in Fixed Analyzer Transmission (FAT) mode using a non-monochromatic Mg source (K_α_ line with hν = 1253.6 eV) at 100 W. C-O bonds of PEGDME were taken as a reference for calibrating the energy scale of the spectra, giving O 1s photoelectron peak at 532.5 eV and C 1s peak at 286. 3 eV, provided that C-C bonds of adventitious carbon appear at 285.0 eV using this calibration. A Shirley function was employed to simulate the inelastically scattered electrons background and Voigt functions (70% Gaussian and 30% Lorentzian) as line-shapes to fit the different photoelectron peaks in the CasaXPS software. Single components were used for the 1s core levels of F, O (not shown), and C and doublets with constrained 2:1 area ratio and 1.18 eV of spin-orbit splitting for the S 2p peak of each specie (splitted in 2p_3/2_ and 2p_1/2_) in the spectra.

In order to complement the physicochemical characterization of the membranes, they were also measured using attenuated total reflectance-Fourier transform infrared (ATR-FTIR) spectrophotometry with a Spectrum 400 DTGS spectrometer (PerkinElmer, Waltham, MA, United States), measuring the infrared spectra in the 400 to 4000 cm^−1^ wavenumber range.

The chemical stability of the GPE membranes with lithium polysulfides was tested by preparing a LiPS solution and depositing it over the pristine GPE electrolytes to evaluate the possible interaction between them. A 0.1 M Li_2_S_6_ solution in PEGDME and TEGDME was prepared by mixing stoichiometric amounts of S_8_ and Li_2_S in the organic solvents and stirring thoroughly for 4 days until an intense-red solution was obtained.

## Data Availability

Not applicable.

## Supplementary Materials

The following supporting information can be downloaded at: https://www.mdpi.com/article/10.3390/gels9040336/s1, Figure S1: The aging test of TEGDME_GPE in contact with Li0 anode.; Figure S2: The aging test of the PMMA/PEGDME_GPE after contact with Li0 anode.; Figure S3: The aging test of PEGDME_LE and TEGDME_LE with Li0 anode after 14 days in contact.; Figure S4: ATR-FTIR spectra of the TEGDME_GPE-based electrolyte before and after being in contact with Li0 anode.; Figure S5: Ionic conductivity at RT of both PEGDME_GPE and TEGDME_GPE and their corresponding liquid electrolyte counterparts; Figure S6: Enlarged symmetrical cell voltage profile data of TEGDME_GPE at different operation times.
