# Supplementary material for: Dehydrofluorination Process of Poly(vinylidene difluoride) PVdF-Based Gel Polymer Electrolytes and Its Effect on Lithium-Sulfur Batteries
Source: Gels. 2023 Apr 14;9(4):336. doi: 10.3390/gels9040336 (PMC10137538; doi:10.3390/gels9040336)
Supplement: Supplementary file 1 [file gels-09-00336-s001.zip › gels-2326330-supplementary.pdf]

Dehydrofluorination Process of Poly(vinylidene difluoride) PVdF-Based Gel Polymer Electrolytes and Its Effect on Lithium-Sulfur Batteries

Julen Castillo [1,2], Adrián Robles-Fernandez [1], Rosalía Cid [1], José Antonio González-Marcos [2], Michel Armand [1], Daniel Carriazo [1,3], Heng Zhang [4] and Alexander Santiago [1,*]

[1] Centre for Cooperative Research on Alternative Energies (CIC energiGUNE), Basque Research and Technology Alliance (BRTA), 01510 Vitoria-Gasteiz, Spain
[2] Department of Chemical Engineering, Faculty of Science and Technology, University of the Basque Country UPV/EHU, Campus de Leioa, Barrio Sarriena, ES-48940 Leioa, Bizkaia, Spain
[3] IKERBASQUE, Basque Foundation for Science, 48013 Bilbao, Spain
[4] Key Laboratory of Material Chemistry for Energy Conversion and Storage (Ministry of Education), School of Chemistry and Chemical Engineering, Huazhong University of Science and Technology, Wuhan 430074, China

* Correspondence: asantiago@cicenergigune.com;

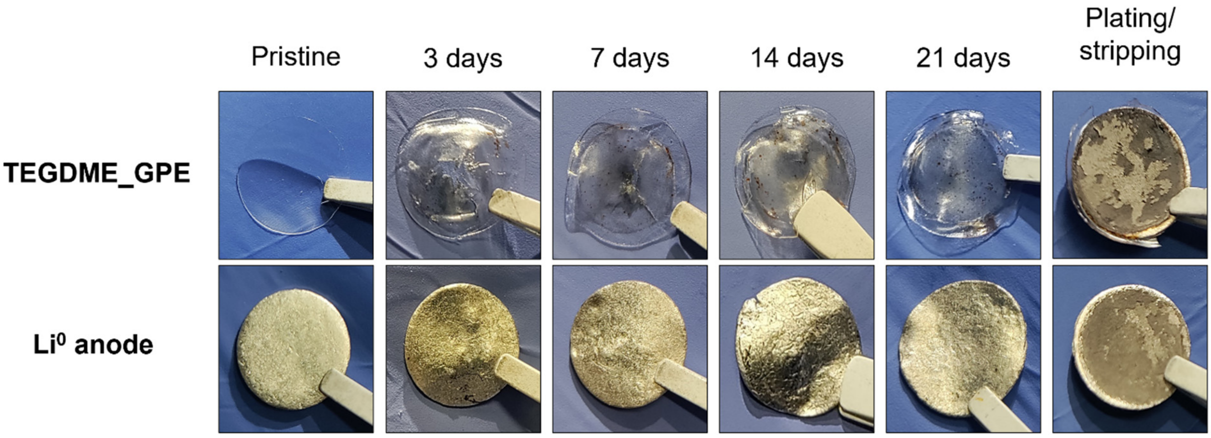

Figure S1. The aging test of TEGDME_GPE in contact with Li^0 anode.

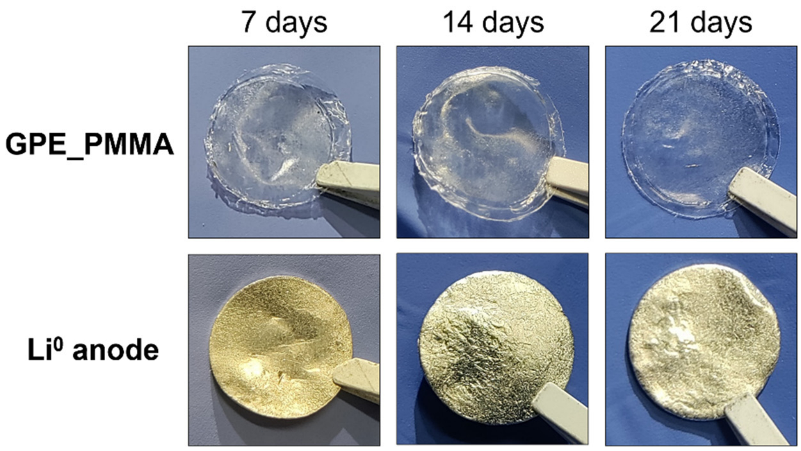

Figure S2. The aging test of the PMMA/PEGDME_GPE after contact with Li^0 anode.

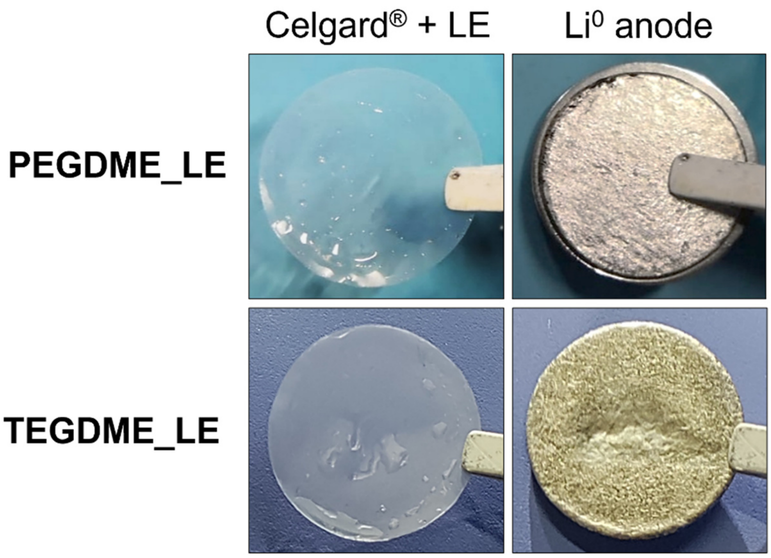

Figure S3. The aging test of PEGDME_LE and TEGDME_LE after 14 days in contact with the Li^0 anode.

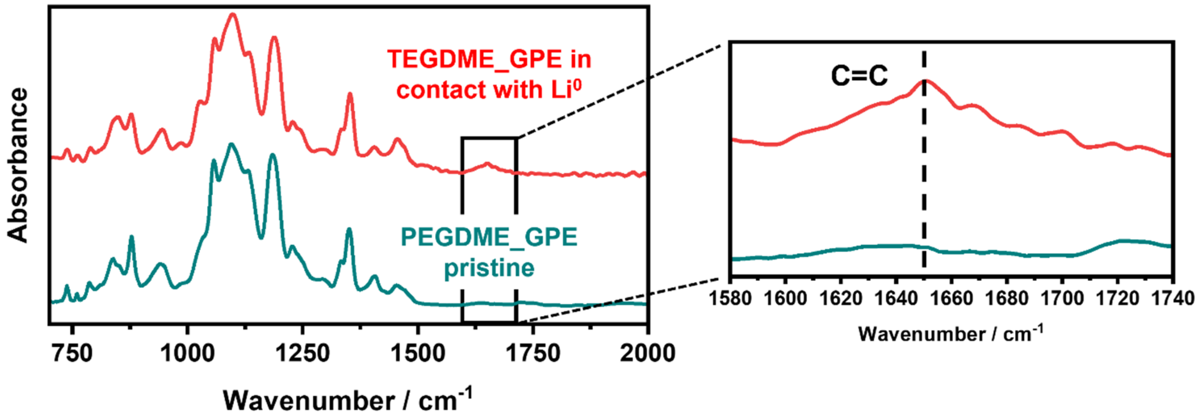

Figure S4. ATR-FTIR spectra of the TEGDME_GPE-based electrolyte before and after being in contact with Li^0 anode.

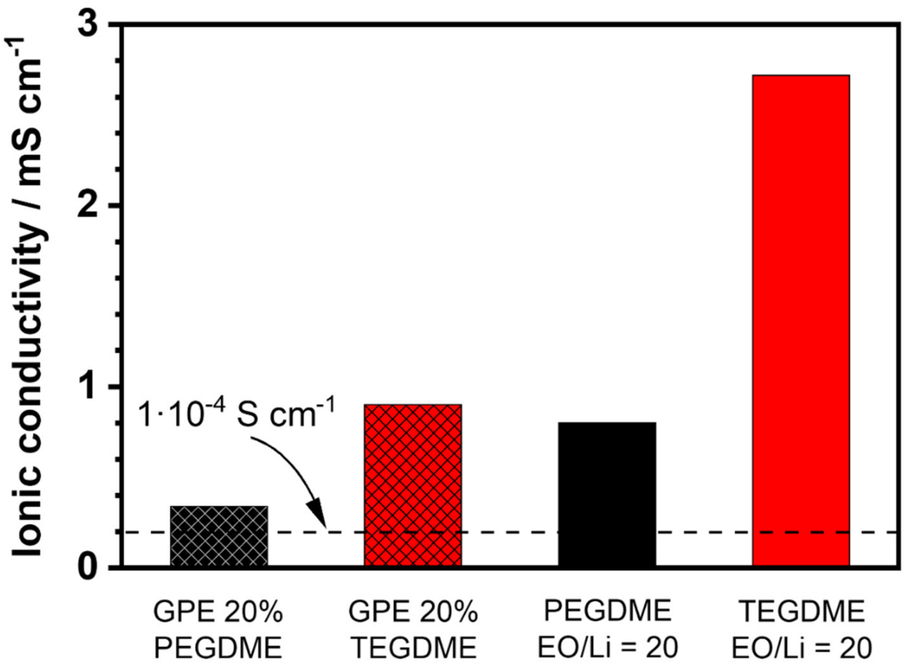

Figure S5. Ionic conductivity at RT of both PEGDME_GPE and TEGDME_GPE and their corresponding liquid electrolyte counterparts.

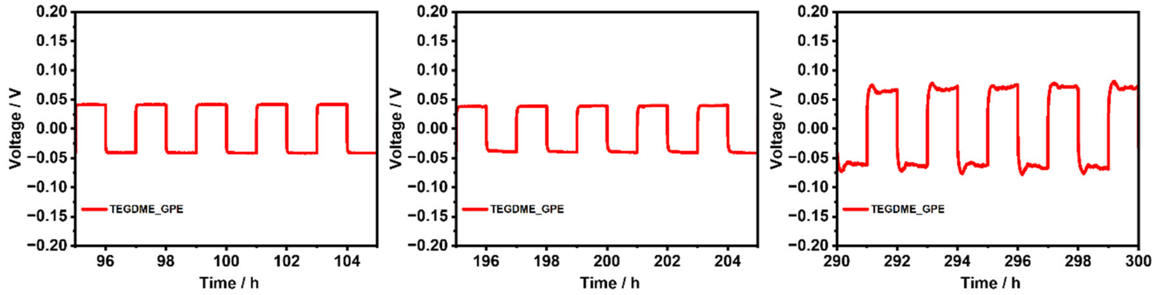

Figure S6. Enlarged symmetrical cell voltage profile data of TEGDME_GPE at different operation times.
